# Cross-Sectional Associations Between Lifetime Use of Psychedelic Drugs and Psychometric Measures During the COVID-19 Confinement: A Transcultural Study

**DOI:** 10.3389/fpsyt.2021.687546

**Published:** 2021-06-16

**Authors:** Dóra Révész, Genís Ona, Giordano N. Rossi, Juliana M. Rocha, Rafael G. dos Santos, Jaime E. C. Hallak, Miguel Á. Alcázar-Córcoles, José C. Bouso

**Affiliations:** ^1^Center of Research on Psychological and Somatic Disorders (CoRPS), Department of Medical and Clinical Psychology, Tilburg University, Tilburg, Netherlands; ^2^ICEERS—International Center for Ethnobotanical Education, Research, and Service, Barcelona, Spain; ^3^Medical Anthropology Research Center (MARC), Universitat Rovira i Virgili, Tarragona, Spain; ^4^Department of Neurosciences and Behavior, Ribeirão Preto Medical School, University of São Paulo, Ribeirão Preto, Brazil; ^5^National Institute of Science and Technology—Translational Medicine, São Paulo, Brazil; ^6^Department of Biological and Health Psychology, School of Psychology, Autonomous University of Madrid (UAM), Madrid, Spain

**Keywords:** psychedelic drugs, distress, social support, psychopathology, personality, COVID−19, cross-cultural

## Abstract

**Background:** One of the main public health strategies adopted at the beginning of the COVID-19 pandemic consisted of implementing strict lockdowns to stop the transmission of the virus. Despite being an effective measure, the confinement and the associated social isolation create a stressful, potentially lengthy situations that has been proven to have several psychological consequences. Given the potential benefits that certain psychedelic drugs have shown for the treatment of psychological disorders, this study aimed to assess the impact of lifetime psychedelic drug use on mental health in relation to the first strict lockdown adopted by various countries (April-July 2020).

**Methods:** Subjects completed an online survey that inquired about sociodemographic factors, activities, and lifestyle factors during confinement, as well as health and mental health related factors. Subjects were asked about their lifetime use of psychedelic drugs (MDMA, ayahuasca, psilocybin-containing mushrooms, LSD, peyote, San Pedro, Bufo alvarius or 5-MeO-DMT, and others), being classified as regular users (more than once per 6 months), occasional users, or non-users. The survey included psychometric tests used to assess psychological distress, peritraumatic stress, social support, psychopathological symptoms, and personality. Linear regressions were performed with psychedelic drug users as the independent variable and psychometric factors as the outcomes, while correcting for age, gender, language, religion, spirituality, and use of non-psychedelic drugs.

**Results:** The study included 2,974 English, Portuguese, and Spanish speakers (497 regular users of psychedelic drugs, 606 occasional users, and 1,968 non-users). On average, respondents were 36 years old and 70% were female. Psychedelic drug users, especially regular ones, reported less psychological distress, less peritraumatic stress, and more social support. Regarding personality measures, psychedelic drug users scored higher on the novelty-seeking and self-transcendence scales, and lower on cooperativeness.

**Conclusion:** Our findings showed that regular users of psychedelic drugs had less psychological stress and some personality differences when compared to occasional users and non-users. This suggests that either the use of psychedelics might be a protective factor itself or people with certain previous traits are more prone to frequently using psychedelic drugs. Future prospective longitudinal research should investigate the underlying processes observed in this study to develop consistent hypotheses.

## Introduction

The new 2019 coronavirus (SARS-COV-2) is a serious and fatal public health concern that has spread globally following the outbreak of the virus between late December 2019 and early January 2020 (1). On March 11th, 2020, the World Health Organization (WHO) officially declared this to be a worldwide pandemic. At the time of this manuscript's preparation (March 2021), the total number of confirmed cases is about 124,535,520 million worldwide, with almost 2,738,876 deaths linked to this virus (2). As this virus is closely related to previous coronavirus outbreaks, such as the severe acute respiratory syndrome (SARS)-CoV and the Middle East respiratory syndrome (MERS)-CoV, the primary symptoms of COVID-19 contraction are generally related to these other respiratory syndromes, including pneumonia, acute respiratory distress syndrome, fever, cough, fatigue, hemoptysis, acute cardiac injury, hypoxemia, dyspnea, and lymphopenia, but also other more specific symptoms, such as rhinorrhea, sneezing, sore throat, and diarrhea (1). The primary dispersion mechanism is believed to be direct contact or droplets spread by infected individuals coughing or sneezing.

Considering the seriousness of this virus and how quickly it spreads, medical experts have advised governments around the world to implement social distancing policies in their countries in order to hamper the virus's transmission. Nevertheless, in addition to the fear of contracting the disease, quarantine causes its own set of problems, particularly associated with mental health and well-being (3–9). Several stressors, including infection fears, frustration, boredom, inadequate supplies, inadequate information, financial loss, and stigma have been found to be correlated with psychopathological symptoms (4, 5) as well as post-traumatic symptoms (4, 10).

Indeed, in late February 2020, the first nationwide large-scale survey of psychological distress in the general population of China during the COVID-19 epidemic was performed (*N* = 52,730) (11). This survey demonstrated that almost 35% of the population was experiencing psychological distress (such as anxiety and depression), with females more affected than males, and people from 18 to 30 and above 60 years of age were more affected than the rest of the population (11). Other surveys showed similar findings (12–15). Health professionals (e.g., doctors, nurses) seem to be at a greater risk of developing mental health problems during the COVID-19 pandemic, including depression, anxiety, insomnia, and distress, as they are constantly exposed to contamination sources in hospitals and are afraid of spreading the virus to family and friends (8, 16–21). Remarkably, adopting a positive coping style has been negatively correlated with anxiety and depression (21). Studies conducted outside of Asia and Europe, while transcultural, reported similar results (22, 23).

Parallel with the pandemic, several studies have been published regarding the potential therapeutic effects of psychedelic drugs, such as ayahuasca, psilocybin, and lysergic acid diethylamide (LSD) (24–27), especially for the treatment of mental health issues (25, 28–30). Other studies have shown their potential for the treatment of pain (31, 32) and neurodegenerative disorders (33, 34). These benefits have also been observed in real-world situations through large population surveys, where psychedelic drug users have been shown to have a lower rate of mental health problems (35, 36), reduced psychological distress and suicidality (37), and better outcomes in terms of internationally-validated health indicators (38). Regarding the latter study, a remarkable result concerned the involvement of complex psychological variables previously unassessed in this population, such as coping strategies and personal values (38). Notably, the benefits associated with psychedelic drugs go beyond clinical outcomes, reaching social aspects. For instance, a relationship between psychedelic drug use and pro-environmental behavior has been found (39), as well as with a lower risk of partner violence (40). Considering these results, some authors have suggested that psychedelic drugs can also play a role in terms of helping individuals face the psychological challenges associated with this pandemic (41).

Given the negative impact of the COVID-19 pandemic on mental health (42), the aim of this study was to explore the potential protective effect of lifetime use of psychedelic drugs through psychometric measures. In order to perform a comprehensive analysis, this research was carried out using a multi-language and transcultural approach, analyzing the associations between psychedelic drug use and psychopathology, peritraumatic stress, and personality. We also investigated other lesser-explored sociodemographic data possibly related to the current pandemic and its associated social distancing measures.

## Materials and Methods

### Setting and Participants

An online survey was launched in three languages (Spanish, English, and Portuguese) on April 7th, 2020, when most countries were moving to implement lockdown measures. Through snowball sampling, researchers from various countries, including mainly Spain and Brazil, spread the questionnaires among direct contacts and through social media. The questionnaire was also shared on the websites of the Mental Health Post-graduate Program of the Ribeirão Preto Medical School at the University of São Paulo, in the scientific journal *Archives of Clinical Psychiatry*, and on websites offering information about psychedelics and cannabis (Lasdrogas.info, Cannabis Magazine, social media pages of ICEERS and local community websites). The three versions of the questionnaire remained open for a period of 6 weeks.

The study was approved by the Research Ethics Committee of the Universidad Autónoma de Madrid (Autonomous University of Madrid, Spain). All experimental procedures were performed in accordance with the relevant guidelines and regulations, and all respondents gave informed consent.

### Psychometric Outcomes

#### General Health Questionnaire–12

The GHQ-12 is a 12-item questionnaire widely used to screen for psychological distress in the general population. The validated Spanish (43), English (44), and Portuguese (45) versions were used. The GHQ-12 consists of 12 items scored using 4-point Likert scales. There are various methods of correction. We used the dichotomous score (0-0-1-1), also called the “GHQ score.” As informed by other studies (46), a score of >4 was established as a cut-off point for considering the presence of mental health disturbance. A higher score represents greater psychological distress.

#### Social Support by the Duke-UNC-11

The Duke-UNC-11 is an 11-item measure of perceived social support. The validated Spanish (47) and English (48) versions were used. The questionnaire was translated to Portuguese by native Portuguese-speaking researchers for the Portuguese version of the questionnaire. The Duke-UNC-11 questionnaire has two dimensions, regarding confidential support (communication with other people) and affective support (expressions of love and affect to/by others). The items are scored using a Likert scale ranging from 1 (“much less than I would like”) to 5 (“as much as I would like). The total score is calculated as the mean of all of the items. The higher the score, the more social support was perceived.

#### Peritraumatic Stress Inventory

The PSI is a proposed measure of post-traumatic stress disorder (PTSD); that is, symptoms associated with the exposure to a potentially traumatic experience. The validated English version (49) was used. The questionnaire was translated into Spanish and Portuguese by native Spanish and Portuguese researchers, respectively. The 13 items of the PSI use a 5-point Likert scale response format, ranging from 0 (“not at all”) to 4 (“extremely true”). The total score is obtained by determining the mean response across all 13 items. Higher scores represent greater exposure to potentially traumatic experiences.

#### Brief Symptom Inventory

The BSI is a brief form of the SCL-90-R questionnaire regarding psychopathology. It is a self-report symptom inventory designed to assess psychological status. The 53-item English validated version (50) was used. For the Portuguese version, the same 53 BSI items were extracted from the Portuguese validated version of the SCL-90-R (51). For the Spanish version of the questionnaire, a similar 45-item validated short version of the SCL-90-R [SA-45; (52)] was used. The three instruments use a 5-point Likert scale ranging from 0 (“not at all”) to 5 (“extremely”). The scales included are the same as those included in the SCL-90-R: somatization (SOM), obsessive-compulsive (O-C), interpersonal sensitivity (IS), depression (DEP), anxiety (ANX), hostility (HOST), phobic anxiety (PHOB), paranoid ideation (PAR), and psychoticism (PSY). Additionally, the general severity index (GSI) was also extracted, with higher scores indicating more severe symptoms.

#### Temperament and Character Inventory-67

The TCI-R-67 is a short version of the TCI-R (53) self-report measure of personality. The Spanish (54) validated version was used. The same 67 items were extracted from the original questionnaire (53) and from the validated Portuguese version (55) in order to use them in the English and Portuguese versions of the questionnaire, respectively. The questionnaire uses a 5-point Likert scale ranging from 1 (“strongly disagree”) to 5 (“strongly agree”). The questionnaire includes four temperamental scales: novelty seeking (NS), harm avoidance (HA), reward dependence (RD), and persistence (PE). It also includes three character scales: self-directedness (SD), cooperativeness (CO), and self-transcendence (ST). Additionally, it includes a validity scale of 5 items. Each score represents the degree to which respondents have particular personality traits.

### Psychedelic and Non-psychedelic Drug Use

Participants were asked about their use of drugs before and during confinement. We included the use of these psychedelic drugs: MDMA, ayahuasca, psilocybin-containing mushrooms, LSD, peyote, San Pedro, *Bufo alvarius* or 5-MeO-DMT, change, and other psychedelics. We described the consumers of psychedelic drugs and compare them to the never-users. Furthermore, we categorized participants as (a) regular users (more than once per 6 months), (b) occasional users (tried it, but do not use it regularly), and (c) never-users.

We also asked for the settings in which participants were using psychedelic drugs: alone, with friends or a partner, at parties or festivals, at rituals or in a therapeutic setting, or microdosing. We only added MDMA to the list of psychedelic drugs when it is used at rituals or in therapeutic settings. We defined non-psychedelic drugs as alcohol, tobacco, cannabis, cocaine, amphetamines, and MDMA when consumed in other contexts.

#### Sociodemographic Data, Religion/Spirituality, and Health Factors

We recorded age, gender (male, female, transgender, or androgynous), the language of the participant (Spanish, English, or Portuguese), and nationality. For each participant, we recorded their partner status (yes/no). We asked for their religion (atheist, agnostic, or religious) and whether they practice their religion. They were asked about whether they felt like a spiritual person or not, and whether they perform any spiritual practices (e.g., prayer, meditation). Each person was asked whether they have any chronic physical or psychological conditions, and housing-related information such as the kind of house they lived in during the confinement or with whom they lived.

#### Preliminary Factors Related to the Confinement Period

As most participants were still in confinement, we will show preliminary analyses of the following factors. We asked whether persons were diagnosed with COVID-19 or showed any COVID-like symptoms, whether their relatives had COVID-19, and whether anyone around them died from this virus. We asked the number of days they spent in confinement, how well they maintained their confinement, and whether they had a house with an outdoor area. We also asked how many hours per week participants spent outdoors, and whether they followed any anti-contagion advice (e.g., wore gloves, masks). We also asked them to rate their well-being and level of discomfort during confinement on a scale from 1 to 10, with 1 indicating a very low level of well-being and no discomfort at all, while 10 indicated excellent well-being and a very high level of discomfort.

Additionally, subjects were asked about their lifestyle and the activities they had been engaging in during confinement (yes/no), including aerobic exercise; martial arts; music and singing; playing videogames; watching pornography; reading; watching TV, movies, series, or COVID-19 related news; weightlifting or bending; yoga; pilates; and meditation. We also assessed changes in diet since confinement, in terms of whether participants were eating better, the same, or worse. We also asked whether they had less libido or sex during this confinement period.

Regarding societal and economic changes, we determined how participants' vision of the economic system and the predominant values of our society (i.e., consumerism, competitiveness, progress) were altered. We asked whether they were now in favor of changes, against changes, or that they did not change in this respect. We asked them whether they had lost their job as a consequence of the COVID-19 crisis or if their income was reduced. We also asked participants if their past psychedelic substance use impacted their ability to manage their confinement.

### Covariates

Age, gender, language, and religion were used as covariates in the linear regression models.

### Statistical Analyses

All variables were described as percentages or means and standard deviations. Baseline sample characteristics of regular, occasional, and never-users of psychedelic drugs were compared using chi-square tests for categorical variables and independent sample *t*-tests for continuous variables. We first did this for the sociodemographic factors, religion and spirituality factors, health factors, and drug use. Then we did the same for all COVID-19-related factors. Then, we made an overview in a figure of how psychedelic substances were rated by participants in terms of helping them to manage their period of confinement better. Next, we stratified for the regular and occasional users. As additional analyses, we compared the means for all psychometric outcomes among the Spanish, Portuguese, and English speakers, using analyses of variances (ANOVA).

Subsequently, we performed linear regression analyses to determine associations between consuming psychedelic drugs (yes/no) and the general health score, peritraumatic stress scores, social support, Brief Symptom Inventory scores, and the personality scales. Next, we ran linear regressions with regular and occasional users vs. the never-users of psychedelic drugs as predictors and psychometric factors as outcomes. For each analysis, we adjusted for age, gender, language, religion, and practitioner of religion. All analyses were conducted using SPSS version 24.0 (IBM Corp., Armonk, NY, USA). After Bonferroni-correction (*p*-value of 0.05 divided by 33), we set the significant *p-*value at 0.002, two-tailed.

## Results

### Sample Characteristics

A total of 2,974 subjects were recruited, with an average age of 36 years, and 29% were male, 70% female, and 1% queer, androgynous, or other genders (Table 1). Of all the participants, 54% completed the questionnaires in Spanish, 23% in Portuguese, and 23% in English. In accordance with these numbers, the largest group of subjects responded from Spain (*N* = 1,191), followed by Brazil (*N* = 652), and the United States (*N* = 265). Most of the sample was single (37.6%), followed by “in a relationship” (29.9%), and married (22.8%). The majority of the participants lived in an apartment or flat (51.3%) or a house with a garden (41.4%), in both cases with more than 70 square meters (62.4%). Only 11.6% of the sample lived alone, while 22% shared a house with their partner, and 21.2% lived with their parents. Only 8.5% of the respondents were taking care of people aged between 60 and 90 years, and 2.3% of the sample was living with someone with special needs.

**Table 1:** Sample characteristics of the total sample, and regular vs. occasional vs. never users of psychedelic substances in the past.

	**Total (*N* = 2,974)**	**Regular users (*N* = 497)**	**Occasional users (*N* = 606)**	**Never users (*N* = 1,868)**	***p*^a^**
**Sociodemographics, N (%)**					
Age [years, mean (SD)]	36.3 (13.3)	40.0 (11.0)	38.5 (12.0)	34.7 (14.1)	**<0.001**
Gender					**<0.001**
Men	852 (28.6)	247 (49.8)	202 (33.7)	403 (21.7)	
Women	2,087 (70.2)	248 (50.0)	392 (65.3)	1,447 (77.8)	
Queer/androgynous/others	18 (0.6)	1 (0.2)	6 (1.0)	11 (0.6)	
Language questionnaire					**<0.001**
English	673 (22.6)	223 (44.9)	205 (33.8)	243 (13.0)	
Portuguese	689 (23.2)	47 (9.5)	70 (11.6)	572 (30.6)	
Spanish	1,613 (54.2)	227 (45.7)	331 (54.6)	1,053 (56.4)	
Partner status	1,565 (52.6)	268 (54.0)	322 (53.2)	974 (52.2)	0.74
**Religion and spirituality, N (%)**					
Religion groups					**<0.001**
Atheist	845 (28.4)	96 (21.8)	185 (33.8)	564 (30.8)	
Agnostic	695 (23.4)	164 (37.3)	194 (35.4)	337 (18.4)	
Religious	1,280 (43.0)	180 (40.9)	169 (30.8)	930 (50.8)	
Practitioner of religion	931 (31.3)	186 (42.7)	176 (33.5)	569 (34.5)	0.003
Spiritual person	1,948 (65.5)	435 (87.5)	451 (74.4)	1,062 (56.9)	**<0.001**
Spiritual practices	1,686 (56.7)	404 (81.3)	397 (65.5)	885 (47.4)	**<0.001**
**Health factors, N (%)**					
Chronic diseases	736 (24.7)	115 (23.1)	150 (24.8)	471 (25.2)	0.63
Mental diseases	670 (22.5)	96 (19.3)	153 (25.2)	421 (22.5)	0.06
**Substance use, N (%)**					
**Non-psychedelic**					
Alcohol	1,684 (56.6)	330 (66.4)	403 (66.5)	950 (50.9)	**<0.001**
Tobacco	681 (22.9)	155 (31.2)	206 (34.0)	320 (17.1)	**<0.001**
Cannabis	1,212 (40.7)	345 (69.4)	438 (72.5)	429 (23.0)	**<0.001**
Cocaine	331 (11.1)	119 (23.9)	174 (28.7)	38 (2.0)	**<0.001**
Amphetamines	266 (8.9)	113 (22.7)	132 (21.8)	21 (1.1)	**<0.001**
**Psychedelic**					
MDMA, ecstasy, molly^b^	701 (23.6)	303 (61.0)	325 (53.6)	73 (3.9)	**<0.001**
Ayahuasca	609 (20.5)	357 (71.8)	252 (41.6)	0	**<0.001**
Magic mushroom	841 (28.3)	418 (84.1)	423 (69.8)	0	**<0.001**
LSD	737 (24.8)	354 (71.2)	383 (63.2)	0	**<0.001**
Other psychedelics	487 (16.4)	296 (59.6)	191 (31.5)	0	**<0.001**
Context of use of psychedelic substances					**<0.001**
Not using anything	1,899 (63.8)	6 (1.8)	25 (5.6)	1,868 (100)	
Alone	65 (2.2)	42 (12.9)	23 (5.2)	0	
With friends or partner	293 (9.8)	91 (28.0)	202 (45.5)	0	
At parties or festivals	60 (2.0)	8 (2.5)	52 (11.7)	0	
Rituals/therapeutic	315 (10.6)	174 (53.5)	141 (31.8)	0	
Microdosing	5 (0.2)	4 (1.2)	1 (0.2)	0	

a *Chi square tests for categorical variables and independent sample T-test for continuous variable;*

b *Is only determined as a psychedelic drug when used for rituals or therapeutic settings. Significant (Bonferroni-corrected) p <0.002 are represented bold*.

Regarding ethnicity, most respondents to the Portuguese questionnaire were “white” (*branca*, 85.2%), and most respondents to the English and Spanish questionnaires were Caucasian (88.5%). Other ethnicities included *preta*/black (3.8% in Brazil and 0.43% for the other questionnaires) or *ind*í*gena*/indigenous (0.5% in Brazil and 2.2% for the other questionnaires). The majority of the sample defined themselves as religious (43.0%), followed by atheists (30%) and agnostics (24.6%). For the Portuguese questionnaire, the majority of respondents were Catholics (38%), followed by agnostics (17%), atheists (13.9%), *Esp*í*rita*/Spiritism (12.3%), and afro-Brazilian (3.5%). Among the total sample, only 35.6% were active religious practitioners, while 65.5% considered themselves spiritual.

A notable percentage of the total sample reported having a physical diseases (28.3%) or mental disorder (31.1%). The most commonly reported physical disorder was asthma (4.7%), and the most commonly reported psychological disorders were anxiety (12.3%), depression (10.4%), and PTSD (1.6%).

There were 1,103 participants (37%) who had used psychedelic drugs, and these included 606 occasional users (20%) and 497 regular users (17%). We considered a regular user to be anyone who reported having used psychedelic drugs more than once per 6 months. Table 1 shows differences in sample characteristics between the never, occasional, and regular users of psychedelic drugs. Regular users were slightly older, more often men, more often English speakers, more often agnostic or religious, practiced religion more often, and they more often considered themselves spiritual or performed spiritual practices. Regular users of psychedelic drugs used more non-psychedelic drugs than the occasional or never users. Also, regular users more often used psychedelic drugs alone or in rituals or therapeutic contexts, while the occasional users used them with friends or at parties and festivals.

### Preliminary Factors Related to COVID-19 Confinement Period

All confinement-related factors are shown in Table 2, both for the entire sample and in terms of comparisons between regular, occasional, and never users of psychedelic drugs. The majority of the sample (90.4%) did not experience COVID-19-related symptoms, while 9.3% experienced symptoms and were not tested, and 0.3% had symptoms with a positive result after testing. Among those who tested positive, 5.1% required health care. Moreover, 17.3% of subjects had a relative who had COVID-19, and 4.7% reported the death of a relative due to COVID-19. Users spent more time outdoors and had more access to an outdoor space. Although the regular users followed fewer of the anti-contagion tips, they reported higher levels of well-being and lower discomfort during the confinement period as compared to the never users of psychedelic drugs. While some activities were performed more often by regular users (e.g., music, singing and yoga, pilates, and meditation), others were performed more often by the never users (e.g., aerobic exercise; watching COVID-related news, TV, movies, and series; and playing videogames). Furthermore, regular users reported having better diets during the confinement period, and a higher libido and more sex. Regular users were also more often in favor of change regarding the economic system or the predominant values of our society, and suffered more from working less and having a decreased income. A high percentage of the sample (80.4%) declared having some personal space in which to relax. Similarly, 61.9% of subjects had a specific space inside the house in which to work. Remarkably, 17% of respondents lost their jobs due to the pandemic, while 47.4% experienced a reduction in their income.

**Table 2:** Preliminary factors related to COVID-19 and the confinement period in the total sample, and in regular vs. occasional vs. never users of psychedelic substances in the past.

***N* (%)**	**Total (*N* = 2,974)**	**Regular users (*N* = 496)**	**Occasional users (*N* = 606)**	**Never users (*N* = 1,865)**	***p***
Diagnosed with COVID-19					0.06
No	2,686 (90.3)	433 (87.1)	545 (89.9)	1,708 (91.4)	
No test, but clear symptoms	275 (9.2)	62 (12.5)	58 (9.6)	155 (8.3)	
Yes, tested positive	10 (0.3)	2 (0.4)	3 (0.5)	5 (0.3)	
Relatives with COVID-19	514 (17.3)	95 (19.2)	108 (17.8)	311 (16.7)	0.40
Deaths for COVID-19	140 (4.7)	25 (5.1)	21 (3.5)	94 (5.0)	0.27
**Confinement, N (%)**					
Days in confinement [mean (SD)]	29.2 (12.5)	30.7 (12.6)	30.9 (13.1)	28.2 (12.1)	**<0.001**
Maintaining confinement					**<0.001**
I go out every day (with dog)	227 (7.6)	78 (15.8)	60 (10.0)	88 (4.7)	
I go out 4–6 days/week	136 (4.6)	36 (7.3)	30 (5.0)	70 (3.8)	
Up to 3 days/week	654 (22.0)	152 (30.9)	162 (26.9)	340 (18.2)	
Only for necessities or not at all	1,943 (65.3)	227 (46.0)	350 (58.1)	1,366 (73.5)	
House with outdoor area	1,274 (42.8)	243 (48.9)	265 (43.9)	766 (41.1)	0.01
Hours/week outdoors [median (IQR)]	2.0 (4)	4.0 (8)	3.0 (6)	2.0 (3)	**<0.001**
Following anti-contagion tips	2,685 (90.3)	420 (84.5)	533 (88.0)	1,731 (92.7)	**<0.001**
Well-being [1–10, mean (SD)]	5.8 (2.1)	6.6 (2.1)	5.9 (2.1)	5.6 (2.1)	**<0.001**
Level of discomfort [1–10, mean (SD)]	5.6 (2.5)	4.6 (2.4)	5.4 (2.4)	5.9 (2.5)	**<0.001**
**Lifestyle during confinement, N (%)**					
Aerobic exercise	1,674 (56.3)	273 (54.9)	325 (53.6)	1,075 (57.5)	0.19
COVID-19 related news	2,531 (85.1)	379 (76.3)	504 (83.2)	1,648 (88.2)	**<0.001**
Martial arts	136 (4.6)	28 (5.6)	42 (6.9)	65 (3.5)	**0.001**
Music and singing	1,026 (34.5)	258 (51.9)	244 (40.3)	524 (28.1)	**<0.001**
Playing videogames	1,005 (33.8)	107 (21.5)	174 (28.7)	723 (38.7)	**<0.001**
Pornography	739 (24.8)	149 (30.0)	179 (29.5)	410 (21.9)	**<0.001**
Reading	2,436 (81.9)	430 (86.5)	529 (87.3)	1,477 (79.1)	**<0.001**
TV, movies or series	2,702 (90.8)	420 (84.5)	552 (91.1)	1,729 (92.6)	**<0.001**
Weight lifting or bending	1,115 (37.5)	179 (36.0)	220 (36.3)	715 (38.3)	0.52
Yoga, pilates or meditation	1,684 (56.6)	404 (81.3)	452 (74.6)	827 (44.3)	**<0.001**
Change in diet					**<0.001**
Better	1,303 (43.8)	259 (52.1)	287 (47.4)	756 (40.5)	
Same	1,166 (39.2)	187 (37.6)	227 (37.5)	752 (40.3)	
Worse	503 (16.9)	51 (10.3)	92 (15.2)	360 (19.3)	
Less libido or sex	1,448 (48.7)	218 (43.9)	280 (46.2)	950 (50.9)	0.01
**Societal and economic changes, N (%)**					
Change in values of society					**<0.001**
Yes, in favor of change	841 (28.3)	195 (39.2)	188 (31.0)	458 (24.5)	
Yes, against change	948 (31.9)	128 (25.8)	201 (33.2)	619 (33.2)	
No changes	1,180 (39.7)	174 (35.0)	217 (35.8)	789 (42.3)	
Working less					**<0.001**
No	2,313 (77.7)	333 (67.4)	438 (73.2)	1,541 (82.9)	
Yes (or reduced hours)	595 (20.0)	154 (31.2)	149 (24.9)	292 (15.7)	
Retired	44 (1.5)	7 (1.4)	11 (1.8)	26 (1.4)	
Income decreased	1,404 (47.2)	266 (53.6)	314 (52.2)	824 (44.3)	**<0.001**

*Significant (Bonferroni-corrected) p < 0.002 are represented bold*.

Similarly, the self-rated well-being of participants (on a scale ranging from 1 to 10) decreased significantly, as they rated their well-being before the lockdown with a mean of 7.3, while the current rating was 5.8. The rating of their house environment decreased as well, but to a much lesser degree (7.6 vs. 7.2). Regarding confinement measures, most of the sample went outside only to buy food or medicines (52.5%), or they were completely locked down (13.5%). Indeed, 90% of the sample was in confinement for at least 20 days. Accordingly, 61% of the sample noted that they did not normally go out, leaving home only out of necessity. Further, 43.8% of the subjects reported eating healthier since the beginning of confinement measures, while nearly half of the sample (48.7%) noted a reduction in their libido. Most of the subjects rated the quality or reliability of the information about COVID-19 received from either politicians or the media as mostly poor (4 and 4.4, respectively).

Regarding drug use during confinement, 51.3% of subjects used alcohol, 23.1% cannabis, 21.6% tobacco, 4.9% psilocybin-containing mushrooms, 3.3% LSD, 2.4% ayahuasca, 2.1% ecstasy, and 1.5% cocaine, among other drugs. Notably, there were more subjects who reported using fewer drugs than usual during confinement (18.7%) than those who reported using more than usual (13.4%). The most remarkable increase was in the case of alcohol, with 3.9% of the sample drinking more during confinement, followed by cannabis (2.3% of the sample). Only 0.9% of subjects reduced their alcohol use.

### Psychometric Measures Among English, Spanish, and Portuguese Speakers

Most of the countries were under strict lockdowns when the questionnaire was launched, with the remarkable exception of Brazil. This information is relevant when considering differences across countries and/or cultures. Indeed, respondents to the Portuguese questionnaire showed a higher mean score in the GHQ-12 questionnaire, which reflects greater psychological distress (Table 3). In the case of Spanish speakers, they showed significantly higher scores for peritraumatic stress than the English speakers (*p* = 0.001), but not than the Portuguese. Spanish speakers also showed significantly higher scores on the psychopathology scales for somatization and depression. The score for perceived social support provided by the Duke-UNC-11 questionnaire was almost the same across the three questionnaires.

**Table 3:** Comparing means of psychometric measures among Spanish, Portuguese, and English speakers.

	**Portuguese [mean (SD)] *N* = 689**	**English [mean (SD)] *N* = 672**	**Spanish [mean (SD)] *N* = 1,610–1,613**	***p*-value**
**Stress and social support**				
Psychological distress	3.56 (3.60)	3.13 (3.33)	2.66 (3.35)	**<0.001**
Peritraumatic stress	1.52 (0.75)	1.46 (0.78)	1.57 (0.77)	0.01
Social support	41.29 (9.69)	40.82 (9.59)	41.71 (9.15)	0.11
**Brief symptom inventory scores**				
Somatization	0.64 (0.75)	0.62 (0.70)	1.21 (1.10)	**<0.001**
Obsessive-compulsive	1.45 (0.93)	1.37 (0.96)	1.30 (0.98)	**0.002**
Interpersonal sensitivity	1.16 (0.98)	1.13 (0.99)	1.02 (0.97)	**0.002**
Depression	1.37 (0.97)	1.31 (1.00)	1.56 (1.08)	**<0.001**
Anxiety	1.30 (0.93)	1.03 (0.90)	1.34 (1.02)	**<0.001**
Hostility	0.95 (0.81)	0.84 (0.81)	0.71 (1.03)	**<0.001**
Phobic anxiety	1.07 (1.07)	1.15 (0.99)	0.85 (1.20)	**<0.001**
Paranoid ideation	0.88 (0.81)	0.81 (0.78)	0.90 (1.01)	0.12
Psychoticism	0.84 (0.78)	0.79 (0.76)	0.46 (0.75)	**<0.001**
General severity index	0.95 (0.72)	1.15 (0.86)	1.13 (0.80)	**<0.001**
**Personality scales**				
Novelty seeking	23.11 (2.54)	22.65 (3.48)	23.25 (3.91)	**0.001**
Harm avoidance	23.64 (2.86)	23.70 (3.57)	20.39 (4.37)	**<0.001**
Reward dependence	21.36 (2.78)	21.02 (3.38)	23.09 (3.37)	**<0.001**
Persistence	26.15 (3.22)	26.03 (3.10)	27.74 (6.13)	**<0.001**
Excitability	18.38 (2.42)	18.75 (2.40)	17.49 (2.85)	**<0.001**
Self-directedness	24.07 (3.98)	23.38 (3.77)	17.94 (6.06)	**<0.001**
Cooperativeness	23.48 (3.09)	22.10 (4.04)	20.45 (3.45)	**<0.001**
Self-transcendence	24.91 (3.22)	25.12 (3.40)	22.14 (9.24)	**<0.001**

*Significant (Bonferroni-corrected) p < 0.002 are represented bold*.

### Associations Between Use of Psychedelic Drugs and Psychometric Measures

**Figure 1:**
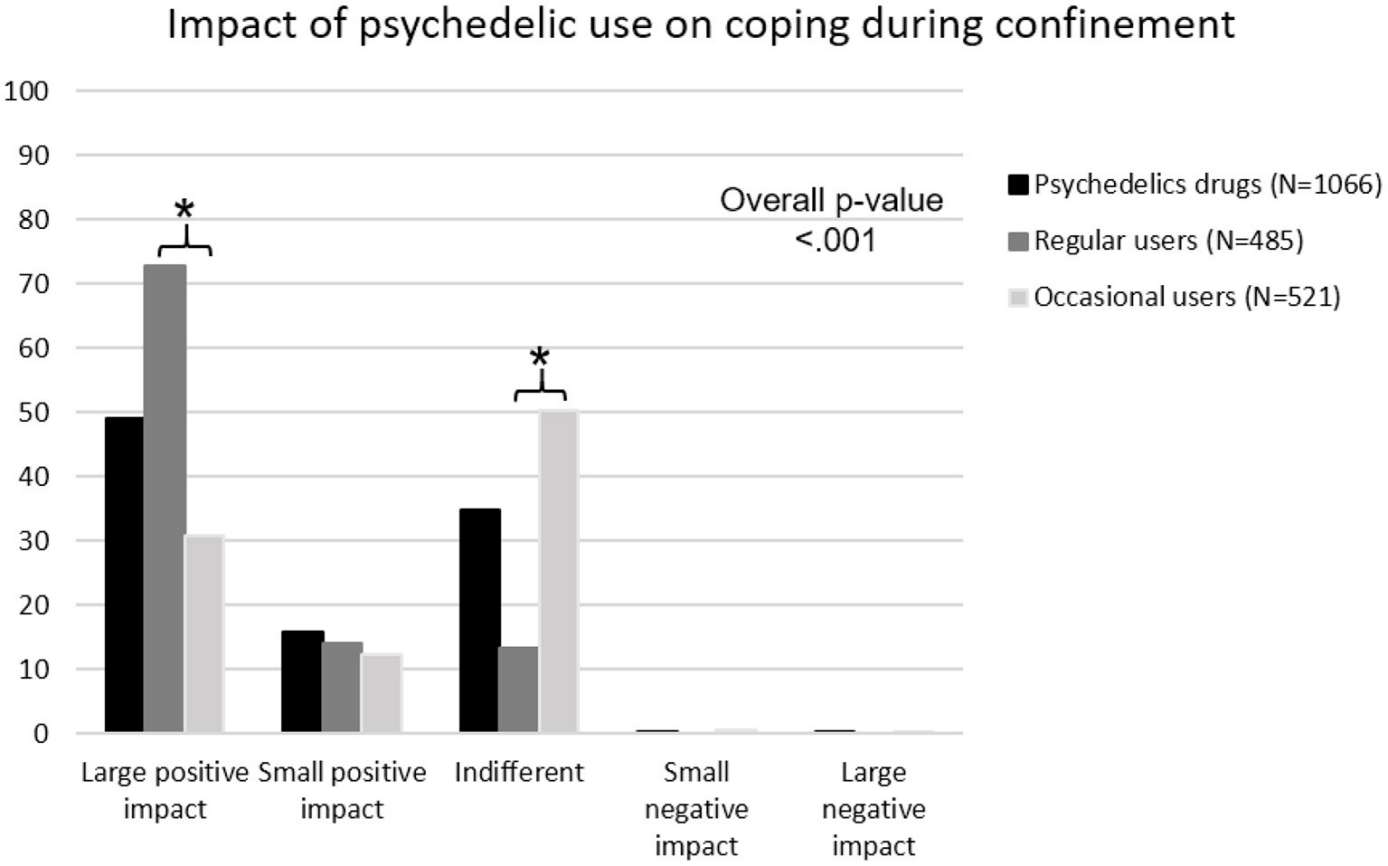
Self-rated impact of psychedelic drug use on managing the COVID-19 confinement period. Black bar refers to persons that have never taken psychedelic drugs; dark gray bar refers to the regular users; and light gray bar refers to occasional users. Overall *p*-value is calculated with Chi square test, and * mark significances between regular and occasional users with *p* < 0.05.

Among the users of psychedelic drugs, 49% reported that their past drug use had a large positive impact on how they managed to cope with the confinement period, while 16% reported that it had a small positive impact, and 35% did not notice a difference (Figure 1). Among the regular users of psychedelic drugs, 73% saw a large positive impact, while only 31% of occasional users reported a large positive impact.

Lifetime use of psychedelic drugs was associated with less psychological distress (Table 4). Lastly, having used psychedelic drugs was associated with higher scores for the novelty seeking and self-transcendence scales in the TCI-R-67 test, but lower scores for cooperativeness. When we examined the frequency of psychedelic drug consumption, regular users had lower psychological distress and peritraumatic stress scores and more social support, while occasional users only showed lower peritraumatic stress (Table 4). Among the personality traits, both occasional and regular users had higher scores for self-transcendence, while only regular users reported more novelty seeking and less cooperativeness.

**Table 4:** Baseline associations between lifetime psychedelic drug users vs. non-users and more specifically, regular vs. occasional vs. never users of psychedelic drugs in the past and psychometric measures.

	**Psychedelic vs. never**		**Occasional vs. never**		**Regular vs. never**	
	**B (SE)**	***p***	**B (SE)**	***p***	**B (SE)**	***p***
**Stress and social support (*****N*** **=** **2,467–2,468)**						
Psychological distress (GHQ)	−0.52 (0.18)	0.004	−0.32 (0.21)	0.12	−0.79 (0.23)	** <0.001**
Peritraumatic stress	−0.17 (0.04)	** <0.001**	−0.16 (0.05)	**0.001**	−0.18 (0.05)	** <0.001**
Social support (Duke 11)	1.53 (0.51)	0.003	1.03 (0.57)	0.07	2.19 (0.63)	** <0.001**
**Brief symptom inventory scores (*****N*** **=** **2,468)**						
Somatization	0.01 (0.05)	0.81	0.03 (0.06)	0.56	−0.02 (0.06)	0.80
Obsessive-compulsive	−0.05 (0.05)	0.30	−0.05 (0.06)	0.42	−0.06 (0.06)	0.33
Interpersonal sensitivity	0.01 (0.05)	0.88	0.01 (0.06)	0.81	−0.00 (0.07)	0.99
Depression	−0.08 (0.06)	0.15	−0.06 (0.06)	0.36	−0.11 (0.07)	0.12
Anxiety	−0.05 (0.05)	0.38	0.00 (0.06)	0.99	−0.11 (0.07)	0.10
Hostility	−0.06 (0.05)	0.28	−0.03 (0.06)	0.62	−0.09 (0.06)	0.15
Phobic anxiety	−0.14 (0.06)	0.03	−0.10 (0.07)	0.16	−0.19 (0.08)	0.02
Paranoid ideation	−0.02 (0.05)	0.69	0.04 (0.06)	0.54	−0.10 (0.06)	0.13
Psychoticism	−0.05 (0.04)	0.28	−0.03 (0.05)	0.58	−0.07 (0.05)	0.16
General severity index	−0.01 (0.04)	0.86	−0.02 (0.05)	0.63	0.01 (0.05)	0.79
**Personality scales (*****N*** **=** **2,467)**						
Novelty seeking	0.75 (0.18)	** <0.001**	0.62 (0.21)	0.003	0.93 (0.23)	** <0.001**
Harm avoidance	−0.10 (0.21)	0.62	−0.14 (0.24)	0.55	−0.05 (0.26)	0.84
Reward dependence	0.08 (0.18)	0.64	0.07 (0.20)	0.72	0.10 (0.22)	0.65
Persistence	−0.18 (0.27)	0.49	−0.37 (0.30)	0.22	0.07 (0.33)	0.83
Excitability	0.12 (0.14)	0.39	0.14 (0.16)	0.37	0.09 (0.18)	0.61
Self-directedness	−0.22 (0.28)	0.44	−0.10 (0.32)	0.75	−0.37 (0.35)	0.28
Cooperativeness	−0.63 (0.19)	**0.001**	−0.29 (0.21)	0.17	−1.09 (0.23)	** <0.001**
Self-transcendence	2.26 (0.36)	** <0.001**	1.89 (0.41)	** <0.001**	2.75 (0.44)	** <0.001**

*All linear regressions were corrected for age, gender, languages, religion, practitioner of religion, alcohol, tobacco, cannabis, cocaine and amphetamine use. Significant (Bonferroni-corrected) p < 0.002 are represented in bold*.

## Discussion

For this study, a large sample was recruited from different countries and cultures and comprehensively assessed in terms of sociodemographic, activity, and lifestyle factors during the confinement, health-related factors, lifetime psychedelic drug use, and psychometric questionnaires. Most of the subjects recruited were in the unique situation of being confined at home due to COVID-19, a very unusual scenario in recent history. We were interested in assessing the potential psychological consequences of such an uncommon social scenario and, more specifically, the possible role that lifetime use of psychedelic drugs might play in dealing with those consequences, given the recently reported clinical and community benefits associated with psychedelic drugs (41). We found that lifetime use of psychedelic drugs, especially when used regularly (defined as at least once per 6 months), was associated with less psychological distress, less peritraumatic stress, and more social support. Regarding personality measures, psychedelic drug users scored higher on the novelty seeking and self-transcendence scales, and lower on cooperativeness.

Regular psychedelic drug users scored better on several psychometric measures. There are various potential explanations for our finding. For instance, this could be related to the better mental health measures that have been found among psychedelic drug users (35–37). Moreover, other survey studies informed about an increasing trend in the use of psychedelic drugs, particularly for self-treatment of mental health conditions (56). The intensity of the so-called mystical-type experience induced by these substances has also been associated with a higher change in well-being (57). It should be noted that psychedelic drug users (both regular and occasional) had various confinement-related differences. For example, they were confined more days than never-users, but the difference, while statistically significant, might not be relevant (a mean of 30.7 and 30.9 days in the case of users, and 28.2 in never-users). A more clearly relevant difference regards time spent outdoors and access to outdoor spaces, where psychedelic drug users had more access. This is in accordance with the enhancement observed in relating to nature after using psychedelic drugs (39, 58, 59). Psychedelic drug users also showed more interest in having healthy habits, such as maintaining a better diet during confinement. This was also found in a previous study with long-term ayahuasca ceremony participants (38), suggesting that beyond the plant/drug itself, other factors might contribute to the generally better health found among this population. Psychedelic drug users also showed higher levels of well-being and lower discomfort levels during confinement, in contrast with the never users. This finding might be linked to the reality that 49% of users self-rated their past psychedelic drug use as having a positive impact on their management of lockdown, while 16% considered it to have a small positive impact. Interestingly, the percentage of people who noted a strong positive impact in Figure 1 is larger among regular users (73%) and reaches only the 31% among occasional users. This may suggest that regular users believe that psychedelic drugs offer greater benefits, or that those supposed benefits are observed only after sustained use. Another potential explanation is that the sample may be biased, being only those who have a strong positive impact the ones who use psychedelic drugs regularly. Moreover, the more psychedelic drugs participants used, the more likely they were to be religious practitioners or define themselves as spiritual and/or performing spiritual practices. These tendencies are highly related to the self-transcendence personality trait (60), which was also measured by the TCI-R-67 test and, accordingly, showed higher scores among psychedelic drug users (both regular and occasional). Regarding religious/spiritual tendencies, previous studies (61, 62) in which personality traits were measured by the TCI/TCI-R-67 questionnaires, involving both long-term ayahuasca ceremony participants and non-users controls, found significant differences for the self-transcendence scale and no differences for the cooperativeness scale. Bouso et al. (61) study was performed with Brazilian ritual ayahuasca users living in urban and jungle areas, whereas the sample for Bouso et al. (62) consisted of Spanish people living in urban areas. The lower score for cooperativeness obtained from psychedelic drug users in our study could be related to differential effects between ayahuasca and psychedelic drugs in general, as well as being affected by participants belonging to formal religious groups, in the case of the samples used by Bouso et al. (61, 62). Interestingly, in another study with Spanish ayahuasca-naïve subjects (28), the score for self-transcendence increased through the follow-ups until 6 months after ayahuasca intake, whereas the score for cooperativeness did not. Although this was not statistically significant, it suggests that having an intense psychedelic experience for the first time can potentiate this trait, and thus the higher scores observed among long-term users can be partially regarded as a consequence of their habits and not due only to previous personality patterns. It should be noted that Bouso et al. (62) found associations between greater self-transcendence scores and increased cortical thinning of the posterior cingulate cortex in the ayahuasca group. The provision of a potential neural basis for this character dimension, and the fact that subjects could experience increased self-transcendence after starting to use psychedelic drugs, supports the hypothesis that this trait is especially notable among long-term psychedelic drug users.

Other factors that explain the better scores obtained by psychedelic drug users might include the physical activity of regular users in our sample. Indeed, psychedelic drug users reported playing music, singing, and doing yoga, pilates, or meditation during confinement, while non-users spent more time watching COVID-related news or TV in general. Some research has warned about inappropriate media exposure during the current pandemic (63), so this may have affected the non-users included in our sample. Additionally, regular users also reported having improved their dietary habits during the lockdown, and having a higher libido and more sex than non-users. However, as a counterpart, they suffered more from working less and having a reduced income. The reasons why the regular use of psychedelic drugs might be protective during a period of confinement can only be speculative. First, they are 5-HT_2A_ agonists and have interesting anti-inflammatory, plasticity-promoting, and neuroprotective properties that could be associated with mood enhancement (34, 64–66). Also, the feelings of awe produced by the psychedelic experience (67) and the enhancement of certain traits or psychological processes, such as decentering (68), can be especially beneficial during a prolonged lockdown. It seems that long-term psychedelic users tend to show these traits (38, 61, 62). This evidence can partially explain some of the differences observed in this study between users and non-users, but further studies should reveal the specific mechanisms at work.

Regular users of psychedelic drugs also reported having used more non-psychedelic drugs in the past. Although drug use is generally associated with poorer outcomes in terms of psychological and physical health, in this case, as we will discuss below, regular psychedelic users scored better on several measures. Notably, we also observed that regular users were more likely using psychedelic drugs alone, in rituals/ceremonies, and in therapeutic contexts, while occasional users used them with friends or at parties and festivals. From a harm reduction perspective, it has been observed that ritualistic and therapeutic contexts are generally safer (69–71). Thus, it might be suggested that regular users tend to choose healthier approaches to drug use, whether psychedelic or not, and, as a consequence, they are less likely to experience negative outcomes.

Our sample was gender-biased (70% women), and was composed of mostly Spanish speakers (54%), mostly from Spain but also from various South American countries. Thus, the results should be interpreted in light of these characteristics. Additionally, it should be noted that the sample was confined for only 20–30 days at the time when they responded to the questionnaire. The lockdown was quite strict in most cases, and these measures lasted much longer than 30 days, so longitudinal studies should discuss the impact of prolonged lockdown in order to appropriately assess the consequences for both physical and psychological health. While most of the sample was under strict lockdown measures, the exception of Brazil is remarkable. Some state and municipal Brazilian governments had implemented certain social distancing measures, whereas the central government opposed such measures (72). In this context, we should ask about the degree of distress that the absence of sensible public health policies can cause, and if such distress is similar to or even greater than the stress caused by the strict lockdowns found in most of countries. Indeed, the GHQ-12 score was significantly higher among the respondents to the Portuguese questionnaire, a finding that supports other data recently published (73). Moreover, according to our results, the feeling of untrustworthiness with regards to politicians and the media was generalized among the entire sample. The reasons underlying this issue are beyond the scope of this manuscript, but due to our social responsibility as scientists we cannot dismiss the opportunity to encourage social scientists, philosophers, and policymakers to analyze and confront this unfortunate social phenomenon.

The most remarkable difference between different versions of the questionnaire and psychopathology measures was observed in the case of Spanish speakers, who scored significantly higher than the other speakers on the somatization and depression scales. This is in line with a recent study (74) that analyzed several variables related to the mental health impacts of the COVID-19 crisis, in which it can be observed that the percentage of the population using benzodiazepines doubled during the lockdown (5.8% of the Spanish population was prescribed benzodiazepines as a consequence of the crisis). The high scores that were observed for some scales (depression and obsessive-compulsiveness) were consistently higher across all countries. Thus, this might suggest similar sources of distress across cultures that could be related to the well-known stressors associated with a global pandemic (e.g., social isolation, disease, and poverty, among others). Unemployment and poverty could be related to enhanced distress (75, 76). The fact that 17% of our sample recently lost their job and that almost half of the total sample reported reductions in income is worrying, since this suggests potential vulnerabilities in terms of health, while also suggesting enormous costs in terms of the global economy. Nearly half of the sample reported a reduction in their libido as well. This has been observed in various studies with contrasting results. While the same percentage of the sample analyzed by Arafat et al. (77) reported that the pandemic affected their sexual life, no alterations were observed in terms of the psychometric measures. Similarly contradictory findings were reported by Yuksel and Ozgor (78), where they found that sexual desire and frequency of intercourse significantly increased during the pandemic, whereas quality of sexual life decreased. This is a complex matter, and future studies should explore the effects of the pandemic on it in depth.

Non-psychedelic drug use during confinement mainly involved alcohol, cannabis, and tobacco. Remarkably, more people reported a general reduction in drug use (18.7%) than reported increased use (13.4%) during lockdown. Specifically, 3.9 and 2.3% of participants increased their use of alcohol and cannabis, respectively, whereas only 0.9% of participants reported a decrease in their alcohol use. These results are in line with findings reported by the Global Drug Survey (GDS) using a sample of 55,000 people recruited in May-June 2020, which also showed a general increase in alcohol and cannabis use, while a smaller part of the sample reduced their use. The use of other drugs decreased dramatically (79). The increase in alcohol use was also reported in US population by the APA (9). However, a Spanish study (80) found that the risky consumption of alcohol actually decreased during the confinement period. Some studies have warned about the clinical risks that cannabis users may face, since they could be more exposed to contagion and have specific vulnerabilities in terms of respiratory issues (81). However, other studies have suggested that some compounds of cannabis might be beneficial for the treatment of some COVID-19 symptoms (82–86). Moreover, other studies reported that cannabis use had positive effects in terms of helping individuals to cope with stressful situations, effects that could partially explain its benefits with regards to chronic disorders (87). These factors should also be considered in the conducting of a risk-benefit assessment. Another online survey (88) that recruited a sample with a similar size as the present study (*n* = 3,632) did not find an increase in cannabis use during lockdown, but the increase in alcohol use was consistent with this study's findings.

This study has some limitations that should be discussed. First, despite the large sample size, the proportion of Spanish speakers was higher than the proportion of English and Portuguese speakers, so the results might not be equally representative for all of these populations. Additionally, regular users of psychedelic drugs were not matched with non-users for some characteristics (e.g., age, gender, and religion). It should be noted that the impact of confinement is difficult to measure, as it includes several aspects that have been only marginally addressed in the recent history of psychology and psychiatry. Thus, the real impact of the pandemic might not be fully reflected in the measures used. Despite our intention to examine the pandemic using a transcultural approach, the psychometric scores obtained should be compared to the normative data for each country to ensure their appropriate interpretation. This is highly challenging since our sample included a large number of nationalities, so we encourage caution when comparing scores between different nationalities without considering intrinsic cultural differences. Lastly, apart from the main interest of this manuscript (i.e., the influence of psychedelic drug use on stress associated with the pandemic), we tried to address some preliminary factors involved in general health and well-being, ranging from self-ratings of discomfort, housing, or family issues, to activities performed during the lockdown. Nevertheless, we are aware of the complexity of the variables we are studying, so we must clarify that other unassessed variables could have influenced the results obtained. Furthermore, when classifying our sample in regular/occasional/never users we should not forget that maybe our results reflect more the characteristics of the people who use psychedelic drugs frequently or not rather than the effects of psychedelic drugs. It should be noted that in the analyses performed in this manuscript it was not taken into consideration whether subjects were living alone or not during the confinement period. Nevertheless, this study has several strengths, such as its large sample size, its precise timing during the first lockdown and related confinement, and the inclusion of persons from various cultures.

## Conclusion

The global pandemic caused by SARS-COV-2 has had a notable impact on several countries across the globe. The consequences go far beyond the virus itself, given the necessary measures for controlling the spread of the virus, which generally consist of limiting social contact. Our sample showed some clear and remarkable signs of psychological distress, and several variables have been analyzed. We found that psychedelic drug use, mostly when used regularly, was associated with better outcomes in terms of general health and regarding stress measures. However, given the observational nature of the design of this study, other factors than psychedelic drug use (e.g., previous personality traits, local lockdown measures, or differences in sociodemographic factors) could be involved in this outcome. This suggests that the use of psychedelic drugs might be a protective factor itself, or people with better psychometric characteristics are more prone to frequently using psychedelic drugs. Therefore, future studies should investigate the different roles that psychedelic drugs can play in this pandemic or in a future pandemic outbreak.

## Data Availability Statement

The raw data supporting the conclusions of this article will be made available by the authors, without undue reservation.

## Ethics Statement

The studies involving human participants were reviewed and approved by Comité ético de la investigación (Universidad Autónoma de Madrid). The patients/participants provided their written informed consent to participate in this study.

## Author Contributions

JB conceived the study. JB, GO, MA-C, RdS, and JH designed the study. GO developed the online survey. GO, GR, and JR performed the data collection. DR performed the statistical analyses. GO, GR, JR, and DR wrote the first draft of the manuscript. All authors contributed to the final version of the manuscript.

## Conflict of Interest

The authors declare that the research was conducted in the absence of any commercial or financial relationships that could be construed as a potential conflict of interest.
